# Expression of somatostatin receptors in hemangioblastomas associated with von Hippel-Lindau disease as a novel diagnostic, therapeutic, and follow-up opportunity: A case report and literature review

**DOI:** 10.20945/2359-4292-2023-0181

**Published:** 2024-05-17

**Authors:** Eloá Pereira Brabo, Sergio Altino de Almeida, Patrícia Piazza Rafful, Paulo Henrique Rosado-de-Castro, Leonardo Vieira

**Affiliations:** 1 Universidade Federal do Rio de Janeiro Hospital Universitário Clementino Fraga Filho Serviço de Oncologia Rio de Janeiro RJ Brasil Serviço de Oncologia, Hospital Universitário Clementino Fraga Filho, Universidade Federal do Rio de Janeiro, Rio de Janeiro, RJ, Brasil; 2 Instituto D'Or de Pesquisa e Ensino Grupo de Oncologia D'Or Rio de Janeiro RJ Brasil Grupo de Oncologia D'Or, Instituto D'Or de Pesquisa e Ensino (IDOR), Rio de Janeiro, RJ, Brasil; 3 Instituto D'Or de Pesquisa e Ensino Departamento de Radiologia Rio de Janeiro RJ Brasil Departamento de Radiologia, Instituto D'Or de Pesquisa e Ensino (IDOR), Rio de Janeiro, RJ, Brasil; 4 Universidade Federal do Rio de Janeiro Departamento de Radiologia Rio de Janeiro RJ Brasil Departamento de Radiologia, Universidade Federal do Rio de Janeiro, Rio de Janeiro, RJ, Brasil; 5 Universidade Federal do Rio de Janeiro Instituto de Ciências Biomédicas Rio de Janeiro RJ Brasil Instituto de Ciências Biomédicas, Universidade Federal do Rio de Janeiro, Rio de Janeiro, RJ, Brasil; 6 Universidade Federal do Rio de Janeiro Hospital Universitário Clementino Fraga Filho Departamento de Clínica Médica e Serviço de Endocrinologia Rio de Janeiro RJ Brasil Departamento de Clínica Médica e Serviço de Endocrinologia, Faculdade de Medicina, Universidade Federal do Rio de Janeiro, Hospital Universitário Clementino Fraga Filho, Rio de Janeiro, RJ, Brasil

## Abstract

Hemangioblastomas associated with von Hippel-Lindau (VHL) disease are frequently multiple and recur during prolonged follow-up. Currently, no systemic treatment is available for these tumors. Recent studies have shown the expression of somatostatin receptors in these types of hemangioblastomas. Notably, increased somatostatin receptor expression in a tumor, as determined by peptide-receptor radionuclide imaging, is a predictive factor of response to treatment with somatostatin analogs and peptide-receptor radionuclide therapy. The aim of this study was to describe the case of a patient with increased expression of somatostatin receptors in a suprasellar hemangioblastoma associated with VHL disease and conduct a literature review on somatostatin receptor expression in patients with VHL-associated hemangioblastomas. We describe herein the case of a 51-year-old man with VHL disease who had a suprasellar hemangioblastoma detected on magnetic resonance imaging. Peptide-receptor radionuclide imaging using gallium-68-DOTATOC (^68^Ga-DOTATOC) identified increased expression of somatostatin receptors in the suprasellar hemangioblastoma, along with multiple pancreatic neuroendocrine tumors and bilateral pheochromocytomas. The patient was treated for 1 year with lanreotide, a somatostatin analog. A repeat ^68^Ga-DOTATOC 1 year after starting lanreotide revealed decreased radiotracer uptake by the hemangioblastoma, consistent with a metabolic response. The presence of somatostatin receptors in hemangioblastomas associated with VHL disease is a novel finding. The decreased expression of these receptors after treatment with a somatostatin analog, as described in the present case, positions the somatostatin receptor as a new target for novel diagnostic, therapeutic, and follow-up opportunities in patients with VHL disease.

## INTRODUCTION

Von Hippel-Lindau disease (VHL) is a rare autosomal dominant condition that predisposes patients to the development of benign and malignant tumors. The main tumors found in these patients include hemangioblastomas of the central nervous system, pheochromocytomas, renal cell carcinomas, pancreatic neuroendocrine tumors (PNETs), renal cysts, pancreatic cysts, and endolymphatic cysts. The follow-up of patients with VHL starts at a young age, enabling preclinical tumor diagnosis and improving the chances of a cure. However, many patients are diagnosed late in adulthood, when the disease is usually at an advanced stage. Although some VHL-associated tumors may present a more indolent behavior compared with sporadic ones, they are invariably fatal upon the development of metastatic or locally advanced disease. Even curative surgery for these tumors can potentially lead to substantial morbidity with a detrimental impact on the patient's quality of life. Thus, the treatment and follow-up of patients with VHL remain challenging for physicians and, particularly, for patients and their families. Considering all these factors, treatment options associated with less morbidity and fewer adverse events and complications are urgently needed (1). Here, we present the case of a patient with VHL and a large suprasellar hemangioblastoma that showed a metabolic response to somatostatin analog treatment. We also present a review of the literature on somatostatin receptor expression and its application in the clinical setting of hemangioblastomas associated with VHL disease.

## CASE STUDY

This case report was approved by the local Research Ethics Committee under protocol number 74411423.8.0000.5249. The patient signed a written informed consent form.

**Figure 1:**
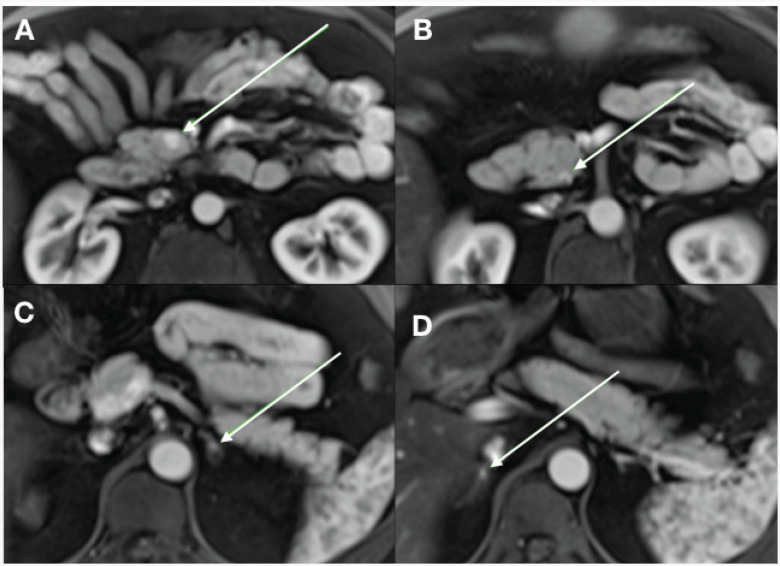
Abdominal magnetic resonance imaging showing two pancreatic neuroendocrine tumors (**A** and **B**) in the presented case and the pheochromocytoma lesions on the left (**C**) and (**D**) right sides.

**Figure 2:**
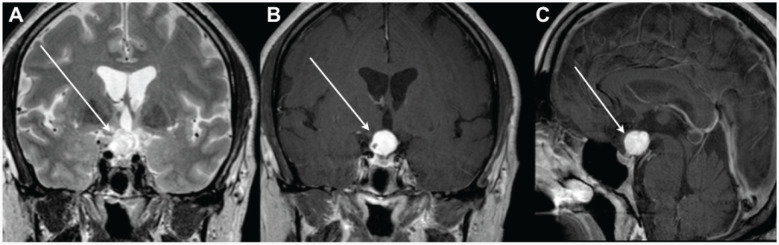
Brain magnetic resonance imaging showing a large suprasellar contrast-enhancing hemangioblastoma. (**A**) T2-weighted image, coronal view; (**B**) T1-weighted image, coronal view; (**C**) T1-weighted image, sagittal view.

The patient was a 51-year-old man with a germline *VHL* missense mutation (c.482G>A) who presented for evaluation in our outpatient clinic. He had undergone laboratory and imaging workup for VHL-associated lesions and an abdominal magnetic resonance imaging (MRI) evaluation that showed the presence of four lesions in the pancreatic head with sizes ranging from 0.3 cm to 1.7 cm (Figure 1). A prior biopsy guided by endoscopic ultrasonography of the largest pancreatic lesion confirmed it to be a grade 1 neuroendocrine tumor. The abdominal MRI also showed bilateral adrenal nodules measuring 0.6 cm and 0.8 cm each (Figure 1) and renal cysts classified as Bosniak I and II. A brain MRI showed a large (1.7 cm) contrast-enhancing suprasellar mass with edema compressing the optic chiasm (Figure 2). Small cerebellar hemangioblastomas and multiple small contrast-enhancing nodules in the dorsal and lumbar spine, suggestive of hemangioblastomas, were also observed. The patient was largely asymptomatic and had no defects on visual field analysis. Laboratory screening revealed elevated 5-hydroxyindoleacetic acid (5-HIAA) level in 24-hour urine (17 mg/24h; normal range < 9 mg/24h), slightly elevated normetanephrine (476 µg/24h; normal range < 450 µg/24h), and minimally elevated prolactin level (21 ng/mL; normal range < 20 ng/mL).

Compared with a previous MRI, obtained 2 years before, the suprasellar lesion was stable. The neurosurgical team evaluated the patient and suggested conservative management instead of resection. The patient refused pancreatic resection at that time.

**Figure 3:**
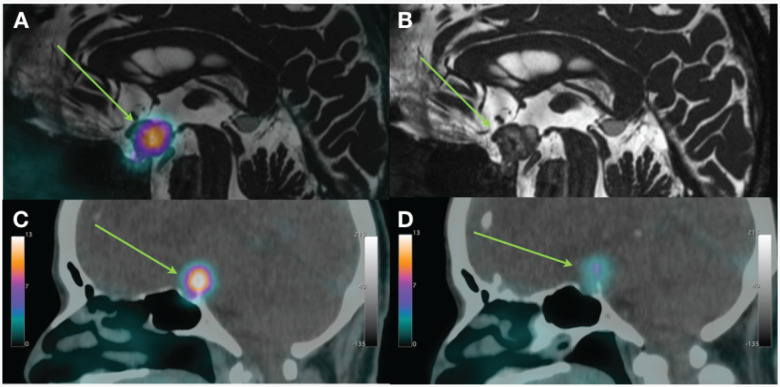
(**A**) Fusion imaging of gallium-68-DOTATOC positron emission tomography –computed tomography (^68^Ga-DOTATOC PET-CT) and brain magnetic resonance imaging showing high uptake by the patient's suprasellar hemangioblastoma, sagittal view; (**B**) brain magnetic resonance imaging of the suprasellar hemangioblastoma without fusion imaging, sagittal view. ^68^Ga-DOTATOC PET-CT imaging of the suprasellar hemangioblastoma (**C**) before lanreotide treatment, sagittal view, and (**D**) after lanreotide treatment, sagittal view.

A gallium-68 DOTATOC (^68^Ga-DOTATOC) positron emission tomography – computed tomography (PET-CT) scan showed high uptake in the pancreatic (maximum standardized uptake value [SUVmax] = 35.4) and suprasellar (SUVmax = 15.6) lesions (Figure 3). Of note, SUVmax is used for semiquantitative analysis of PET-CT images. Lanreotide, a long-acting somatostatin analog, was started at a dose of 120 mg subcutaneously at 28-day intervals, and preoperative evaluation was proposed for the small pheochromocytomas. A few months later, the severe acute respiratory syndrome coronavirus 2 (SARS-CoV-2) pandemic reached Brazil, and the patient continued treatment with the somatostatin analog, which he remained up to the publication of this article. The proposed abdominal surgeries were postponed.

A follow-up ^68^Ga-DOTATOC PET-CT obtained 1 year after the first lanreotide dose showed that the suprasellar and pancreatic lesions did not change in size but had a significant decrease in radiopharmaceutical uptake, respectively, SUVmax = 4.8 (Figure 3) and SUVmax = 24.3.

The patient's living first-degree relatives were screened for the same mutation identified in the patient. The mutation was not present in his mother, and its status in his father, who had died at the age of 50 years of an unknown cause, was undeterminable. One of his two sons had the mutation and was diagnosed with bilateral pheochromocytoma, one at the age of 16 years (right adrenal) and the other at the age of 18 years (left adrenal). He was also diagnosed with PNET at the age of 29 years. The patient's other son did not have the mutation. Renal malignant tumors were not detected in the patient or his son. Therefore, they were both clinically and phenotypically classified as having VHL type 2A – a VHL subtype characterized by the presence of pheochromocytoma but no renal cancer (1).

## REVIEW AND DISCUSSION

We conducted a MEDLINE search using the string "hemangioblastoma and somatostatin receptor." The search retrieved two published case series, seven case reports, and one original study. We also reviewed the references cited in the retrieved papers to search for any missing cases or series.

The first retrieved paper, published in 2008, was a case report of a patient with VHL. The patient underwent indium-111 (^111^In) pentetreotide (Octreoscan) scintigraphy for evaluation of a suspected pancreatic neuroendocrine tumor. The Octreoscan result revealed cerebellar uptake and confirmed the recurrence of a hemangioblastoma (2). The remaining six case reports included patients who underwent PET-CT with gallium-68 DOTA-conjugated peptides (^68^Ga-PET). In two cases, the patients underwent ^68^Ga-PET for evaluation and staging of a PNET and a pheochromocytoma in the context of known VHL disease (3,4). In two other cases, ^68^Ga-PET was performed for staging of a pancreatic tumor in one patient and an adrenal tumor in the other patient; these patients were unaware of the diagnosis of VHL, which was established clinically after the incidental detection of associated hemangioblastomas on ^68^Ga-PET imaging (5,6). In another case, ^68^Ga-PET scanning was performed to evaluate a retinal hemangioblastoma in a patient with an established VHL diagnosis (7). In the last case report, fluorodeoxyglucose PET (^18^F-FDG-PET) and ^68^Ga-PET scanning were both obtained to assess a recent paraparesis in a patient with VHL; a thoracic hemangioblastoma was detected through the ^68^Ga-PET scan but was not identified by the ^18^F-FDG-PET scan (8). Cerebellar or spinal hemangioblastomas were an incidental finding in six of the seven case reports. It is important to note that in one case, somatostatin receptor imaging detected a spinal hemangioblastoma more than 1 year before MRI identification (4).

The first case series retrieved was a cross-sectional prospective study published in 2016. The aim of the study was to investigate the additional role of ^68^Ga-PET for the screening of PNET in patients with VHL (9). The study's follow-up protocol included annual screening evaluations with abdominal, spinal, and brain MRI scans. A total of 20 patients were prospectively examined from January 2012 to November 2015. The ^68^Ga-PET scans detected a greater proportion of PNETs compared with previous examinations using Octreoscan. Additionally, ^68^Ga-PET scans detected somatostatin-receptor-positive cerebellar and spinal hemangioblastomas in three patients. The SUVmax of these hemangioblastomas ranged from 2.1 to 10.1 (9).

The second case series, published in 2019, was a retrospective evaluation of the accuracy of ^68^Ga-PET in detecting pancreatic lesions in 36 patients with VHL. The ^68^Ga-PET scans detected cerebellar hemangioblastomas in five patients (SUVmax was not reported in these lesions) (10).

The only original study retrieved in the search was published in 2017 (11) and assessed the presence of somatostatin receptors in VHL-associated hemangioblastomas and the *in vitro* activity of octreotide. The authors confirmed the histological expression of somatostatin receptors in hemangioblastoma by demonstrating strong expression patterns for all five somatostatin receptors. Nine postsurgical histological samples were evaluated with immunohistochemical analysis to identify the presence of somatostatin receptor subtypes 1 (SSTR1) through 5 (SSTR5). The results indicated that SSTR4 was present in 100% of the samples, while SSTR1, SSTR2, and SSTR5 were found in 89%, and SSTR3 was detected in 22%. All the samples analyzed showed coexpression of at least three somatostatin receptor subtypes. The authors also performed *in vitro* analyses using VHL stromal cell culture to investigate cell viability and apoptosis induction after octreotide exposure and evaluated the role of the canonical hypoxia pathway in octreotide's antitumor effect. The results showed that octreotide decreased cell viability and induced apoptosis and that its antitumor effect did not involve the canonical hypoxia pathway. In the same study, the authors reported the case of a patient with VHL and a large, inoperable, suprasellar hemangioblastoma that responded metabolically and anatomically to long-acting systemic octreotide (11).

Table 1 shows a detailed summary of all the studies retrieved in our literature search.

**Table 1:** Results of a literature review of studies analyzing somatostatin receptor expression in hemangioblastomas associated with von Hippel-Lindau disease

Reference number	Publication year	N	Imaging method	Age	Sex	Hemangioblastoma anatomic location	Somatostatin receptor density
2	2008	1	^111^In-Octreoscan	38	Female	Cerebellar	NR
3	2012	1	^68^Ga-DOTATOC	36	Female	Cerebellar, spinal cord	SUVmax 4.1 and 3.8
5	2014	1	^68^Ga-DOTANOC	52	Female	Cerebellar, retina	SUVmax 9.9 and 8.3
6	2014	1	^68^Ga-DOTANOC	35	Male	Cerebellar	Avid
9	2016	3	^68^Ga-DOTATOC	NR	NR	Cerebellar, spinal cord	SUVmax 2.1-10.1
7	2017	1	^68^Ga-DOTATATE	35	Male	Retina	SUVmax 9.0
11	2018*	1	^68^Ga-DOTATATE	64	Female	Suprasellar	SUVmax 22.6
4	2019**	1	^68^Ga-DOTATOC	42	Male	Spinal cord	Strong
8	2019	1	^68^Ga-DOTATOC	27	Male	Cerebral, spinal cord	SUVmax 10.49, avid
10	2019	5	^68^Ga-DOTATATE	NR	NR	Cerebellar, spinal cord	NR

* The patient was treated with a somatostatin analog and showed decreased SUVmax in a repeat ^68^Ga-DOTATATE 6 months after treatment start.

** ^68^Ga-DOTATOC scanning detected spinal cord hemangioblastomas that were not detected on magnetic resonance imaging. Abbreviations: N, number of patients; NR, not reported; SUVmax, maximum standardized uptake value.

Hemangioblastomas are uncommon tumors of the central nervous system. They are benign vascular tumors of uncertain origin, composed of stromal and endothelial cell components (1). They comprise 1.1%-2.4% of all intracranial space-occupying lesions and typically occur in the cerebellum, brainstem, and spinal cord (12). Approximately one-third of all hemangioblastomas are associated with VHL; in these cases, they are the most common neoplastic manifestations of the disease (12,13). Hemangioblastomas associated with VHL are typically multiple and occur at an earlier age than sporadic hemangioblastomas. The number of hemangioblastomas per patient is on average 10.4 ± 7.8 (median 8). Their anatomical distribution is as follows: cerebellum, 45% of the cases; spinal cord, 37%; cauda equina, 11%; brainstem, 6%; supratentorial, 1%; and nerve roots, 0.3% (13). One of the hemangioblastomas in the present case was located in the suprasellar region. This location strongly suggests association with VHL (12), since sporadic suprasellar hemangioblastomas are extremely rare (14-16).

The treatment of VHL-associated hemangioblastomas depends on the size and location of these lesions and their resulting symptoms. Surgery is recommended for all symptomatic patients and for tumors causing cerebrospinal fluid obstruction (1). However, hemangioblastomas located in critical anatomic areas or in patients with comorbidities, when added to the vascular nature of these lesions, may increase the risk of their complete resection, especially in patients with multiple tumors. In these situations, radiosurgery may be a feasible treatment option, as long-term results with this therapy are now available in patients with sporadic and VHL-associated hemangioblastomas (17-19). Notably, long-term systemic treatments for hemangioblastomas are unavailable (1).

Conservative treatment was recommended for the suprasellar hemangioblastoma in the present case, while a somatostatin analog was initiated for the patient's PNET based on findings from the CLARINET trial (20) and because of the SARS-CoV-2 pandemic, which postponed a planned surgery. A ^68^Ga-PET scan revealed the presence of somatostatin receptors in the patient's hemangioblastomas, and a second ^68^Ga-PET scan reliably confirmed the tumor's metabolic response by showing that the density of somatostatin receptors in the tumor decreased relative to the first scan. To the best of our knowledge, this is only the second report in the literature of this type of response in a VHL-associated hemangioblastoma.

In our literature review, we identified 16 patients with hemangioblastomas expressing somatostatin receptors on Octreoscan and ^68^Ga-PET imaging (2-11). It is important to note that, compared with Octreoscan, ^68^Ga-PET scans have superior spatial resolution, shorter scanning time, and higher sensitivity and specificity. The presence of somatostatin receptors in hemangioblastomas associated with VHL has also been demonstrated in a well-designed study that included clinical and *in vitro* procedures (11). The identification of somatostatin receptors in hemangioblastomas raises many questions about the unknown origin of these tumors and suggests a potential neuroendocrine origin, either associated or not with hemangioblast precursor cells (1).

The presence of somatostatin receptors in hemangioblastomas, particularly the SSTR2 and SSTR5 subtypes, brings to light important considerations about the potential use of these receptors for diagnostic and therapeutic purposes. In diagnostics, ^68^Ga-PET imaging could reveal incidental hemangioblastomas and, in some instances, establish a clinical diagnosis of VHL (5,6). Indeed, ^68^Ga-PET scanning has been reported to identify hemangioblastomas even months before detection of anatomical changes (4). Additionally, ^68^Ga-PET scanning has been reported to show the expression of somatostatin receptors in VHL-associated epididymal cystadenoma (21). The ^68^Ga-PET-measured SUVmax, which reflects the density of somatostatin receptors in the tumor, is a surrogate for the response to treatment with somatostatin analog and peptide-receptor radionuclide therapy (PRRT) (22-24).

Only two cases of somatostatin analog treatment in patients with VHL-associated suprasellar hemangioblastomas have been reported to date, both with favorable results: one described in the original study retrieved in our literature search (11), in which octreotide was used, and the other described in the present report, in which lanreotide was used. While *in vitro* studies have analyzed the octreotide activity in VHL-associated hemangioblastomas, no such studies looking into lanreotide activity have been published. Notably, somatostatin analogs have been used for the treatment of neuroendocrine tumors for more than 30 years; these medications are suitable for long-term use, as they are associated with low morbidity and prolonged efficacy.

For patients with VHL-associated hemangioblastomas who are deemed inoperable and in whom the hemangioblastomas express somatostatin receptors, as determined by ^68^Ga-PET scanning, systemic treatment with a somatostatin analog is a reasonable and safe therapeutic option. This treatment approach has the potential to reduce or delay tumor growth, as described in the two cases reported to date (the original study and the present report) (11). Another treatment option for tumors expressing somatostatin receptors is PRRT. Yet another potential use for ^68^Ga-PET scanning is in the follow-up of patients with VHL, as this method can detect new lesions more accurately and before other imaging modalities (4). These results are clinically promising, although further studies are needed to confirm them. Still, somatostatin analogs emerge as a novel and interesting therapeutic option for the treatment of patients with this devastating disease.
